# Prognosis and Prognostic Factors of Patients with Emergent Cerclage: A Japanese Single-Center Study

**DOI:** 10.1155/2021/4351783

**Published:** 2021-12-26

**Authors:** Ami Kobayashi, Hironori Takahashi, Shigeki Matsubara, Yosuke Baba, Shiho Nagayama, Manabu Ogoyama, Kenji Horie, Hirotada Suzuki, Rie Usui, Akihide Ohkuchi, Hiroyuki Fujiwara

**Affiliations:** Department of Obstetrics and Gynecology, Jichi Medical University, 3311-1 Shimotsuke, Tochigi 329-0498, Japan

## Abstract

**Objectives:**

The aims of this study were to clarify the following: (1) how often does prolonged pregnancy ≥34 weeks occur in patients with emergent cerclage without progesterone and (2) the risk factors preventing such pregnancy continuation.

**Materials and Methods:**

This retrospective observational study was performed using medical records of patients for whom emergent cerclage had been performed between April 2006 and December 2018 in our institute.

**Results:**

Emergent cerclage was performed in 123 patients (median age: 34, interquartile range: 31–36). Primiparous patients numbered 44 (36%). A history of spontaneous preterm birth (SPTB) was present in 30 (24%). The median presurgical cervical length (CL) was 16 (8–21) mm at surgery. Of the 123, 20 (16%) were delivered at 33 + 6 weeks or less (<34 weeks). We conducted logistic regression analysis of the risk factors of SPTBs <34 weeks after cerclage. Three risk factors were identified that increased the risk of SPTB <34 weeks: presurgical CL 0 mm (odds ratio (OR): 5.30; 95% confidence interval (CI): 1.58–17.7), a history of SPTB (OR: 4.65; 95% CI: 1.38–15.7), and the presence of sludge (OR: 4.14; 95% CI: 1.20–14.3).

**Conclusion:**

Three risk factors predicted SPTB <34 weeks after emergency cerclage without progesterone administration: unmeasurable CL (CL 0 mm), a history of SPTB, and the presence of sludge on ultrasound. SPTB <34 weeks occurred after emergency cerclage in 16% of patients, being comparable with the recent data with progesterone.

## 1. Introduction 

Treatment and prophylaxis for spontaneous preterm birth (SPTB) are challenging: administration of progesterone [1], tocolytic agents [2], antibiotics [3], and their combination [4, 5] have been reported to reduce SPTB incidence. Cervical cerclage has also been attempted in some patients. Cerclage is divided into two types: elective and emergent cerclage. The former is performed at 12–14 weeks' gestation for patients with historical indications. The latter, also referred to as rescue cerclage, is usually performed at 15–24 weeks for patients with an ultrasound-detectable shortened cervical length (CL) and patients with this latter cerclage, compared with those with the former clearly show poorer perinatal outcomes, especially PTB. Thus, patients after emergent cerclage require special attention, especially for PTB.

Progesterone has been widely recommended as a treatment for threatened PTB in global guidelines [6–8]. However, in Japan, progesterone is not yet a routine choice because national insurance does not cover it. Without progesterone, we Japanese obstetricians employ cerclage. The fundamental policy of our institute is: emergent cerclage should be performed for a patient with CL <25 mm at <24 weeks of gestation regardless of the presence or absence of a history of SPTB, if contraindications are absent (described later). Nonuse of progesterone gave us a chance to answer the following question: without progesterone, what perinatal outcomes will emergent cerclage yield? We attempted to answer two questions: (1) how often does prolonged pregnancy ≥34 weeks occur (possible effectiveness of cerclage) and (2) what are the risk factors preventing such pregnancy continuation (risk factors of SPTB <34 weeks after cerclage).

## 2. Materials and Methods

This study was approved by the ethics committee of our institute (20–070). We retrieved medical charts of patients for whom cervical cerclage was performed between April 2006 and December 2018 in our institute, in which approximately 1,000 high-risk deliveries are managed annually. Our treatment strategy for SPTB fundamentally adhered to the Japanese guidelines [9–11]. The delivery mode and timing were decided by attending obstetricians.

We performed emergent cerclage in patients with CL <25 mm at less than 24 weeks of gestation irrespective of the presence or absence of a history of SPTB with patients' consent. Transvaginal ultrasound was routinely performed between 19 and 23 weeks of gestation. In high-risk SPTB patients, we measured CL weekly or biweekly from 15 to 24 weeks. Patients referred from other institutes due to shortened CL were also included. We included patients with shortened CL regardless of the presence or absence of preterm labor pains. If CL could not be measured because of cervical effacement and cervical shorting, we defined the condition as CL 0 mm. We did not employ cerclage in the following patients (exclusion criteria): those with (1) active vaginal bleeding, (2) evident chorioamnionitis (e.g., white blood cell ≥15,000/*μ*L, high-grade fever, and fetal tachycardia), (3) prolapse of the membrane (bag) into the vagina, (4) rupture of the membrane, (5) continuous preterm labor pains despite administration of tocolytic agents, (6) lethal major fetal anomalies, and (7) those not consenting to the cerclage. We excluded iatrogenic preterm births including preeclampsia, medical complications, and non-reassuring fetal status before labor pains from this study. We also excluded placenta previa, fetal abnormalities, and multiple deliveries. Amniocentesis to measure subclinical infection was not performed. Although emergent cerclage was performed from 15 to 24 weeks' gestation, it was exceptionally performed at 24 to 26 weeks in a few cases based on patients' wishes and obstetricians' decisions.

Regarding the operative procedure, McDonald cerclage was performed as follows. After spinal anesthesia using ropivacaine or bupivacaine, patients were placed in a lithotomy position. If necessary, the Trendelenburg position was concomitantly employed. Using 1 monofilament nylon thread, we placed single McDonald cerclage as described as a standard technique. We included patients in whom the OS opened 1–4 cm and the membranes were visible: we pushed the membrane back into the uterus (membrane replacement) in some patients using a wet gauze and/or small balloon (Mini-Metro, Soft Medial, Tokyo, Japan). After the operation, systemic antibiotics (e.g., flomoxef sodium) were administered intravenously for more than 3 days. After the operation, the patients were hospitalized for more than one week. Ritodrine hydrochloride, magnesium sulfate, or their combination was administered as tocolysis in all patients. The attending obstetricians decided on the periods of these administrations and hospitalizations. Vaginal or intramuscular progesterone was not administered because it is not covered by national insurance. Vaginal ulinastatin, which is a granulocyte elastase inhibitor, was administered every day based on patients' wishes during hospitalization and also based on the Japanese guideline [9–11]. Blood analyses were performed more than once a week. The cerclage thread was removed at 36 weeks of gestation. When complete premature rupture of the membranes occurred, the thread was removed regardless of gestational weeks. We routinely performed transvaginal ultrasound once a week, using a 5–7.5 MHz transducer.

From electronic medical records, we retrieved maternal backgrounds (e.g., age, parity, mode of conception, smoking, presence of cervical polyp, a history of uterine surgery, a history of cesarean section (CS), and a history of SPTB), CL, presence/absence of sludge on ultrasound, vaginal bacterial culture, and the presence/absence of cervical elastase [12], gestational week at delivery, birth weight, fetal sex, and neonatal intensive care unit admission. Regarding the presence or absence of sludge, it was not familiar to general obstetricians until approximately 2011. Thus, we checked ultrasound images in medical charts in all the included patients. AK and HT checked and judged the presence or absence of sludge separately. If opinions were divided, AK judged it. The Mann–Whitney *U*-test and Fisher's exact test (two-tailed) were used to compare characteristics, maternal backgrounds, and outcomes between deliveries at less than 34 weeks' gestation (+) vs. (−). Parameters significant (*P* < 0.15) on univariate analysis were used for logistic regression analysis. All analyses were performed using JMP Pro version 15 (SAS Institute, Tokyo, Japan), with *P* < 0.05 considered significant.

## 3. Results

Table 1 shows patients' backgrounds. Cervical cerclage was performed in 338 patients during the study period. Of those, emergent cerclage was performed in 123 (median age: 34, interquartile range (IQR): 31–36). Primiparous patients numbered 44 (36%). Pregnancies after assisted reproductive technology (ART) accounted for 13 (11%). Histories of CS, myomectomy, cervical cerclage, and conization were noted in 21, 2, 7, and 2 patients, respectively. A history of SPTB was present in 30 (24%). Slight bleeding at admission was noted in 7 (6%). The median presurgical CL was 16 (IQR: 8–21) mm. The presence of sludge in ultrasound was observed in 24 (16%). Regarding vaginal culture, *Lactobacillu*s and ureaplasma were present in 89 (72%) and 42 (34%), respectively. The prolapsed membrane was replaced by pushing-back at surgery in 22 (18%). No uterine malformation was noted. Premature rupture of the membrane at surgery did not occur in any patients.

**Figure 1:**
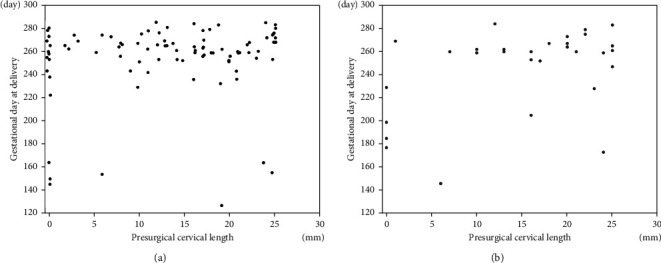
Scatter plot of included patients (123 patients) in both groups without (a) (93 patients) and with (b) (30 patients) a history of SPTB. *x*-axis: presurgical cervical length. *y*-axis: gestational age at delivery. 280 days = 40 + 0 weeks of gestation. 259 days = 37 + 0 weeks of gestation. 238 days = 34 + 0 weeks of gestation. 210 days = 30 + 0 weeks of gestation. 168 days = 24 + 0 weeks of gestation. All cases with both CL = 0 mm and a history of SPTB resulted in SPTB <34 weeks.

Table 2 shows the perinatal outcome of patients with emergent cerclage. Of the 123, 79 (64%) and 44 (36%) resulted in term deliveries and PTBs, respectively: of the latter 44, 20 (16%) were delivered at 33 + 6 weeks or less (<34 weeks). The median birth weight was 2,706 g. NICU admission was required in 51 (41%).  Figure 1 shows a scatter plot of the 123 patients in both groups with or without a history of SPTB. All cases with both CL = 0 mm and a history of SPTB resulted in SPTB <34 weeks.

Table 3 shows a univariate analysis of risk factors of SPTB <34 weeks, obtained by the comparison between patients delivered before vs. after <34 weeks. The former (<34 weeks), compared with the latter (≥34 weeks), had (i) a significantly more frequent CL 0 mm (8 (40%) vs. 12 (12%), respectively; (*P*=0.003) and (ii) significantly more frequent ultrasound-detectable sludge (8 (40%) vs. 16 (16%), respectively (*P*=0.029). Patients with SPTB <34 weeks frequently had a history of SPTB, although it was not significant (8 (40%) vs. 22 (21%), respectively (*P*=0.091). The median duration between the cerclage and discharge showed a shorter tendency in patients with SPTB <34 weeks than those with ≥34 weeks (35 (IQR: 21–84) vs. 76 (IQR: 13–98) days, respectively (*P*=0.057).

Table 4 shows logistic regression analysis of the risk factors of SPTBs <34 weeks after cerclage. Long-term tocolysis and hospitalization management were employed for the majority of patients in this study. Thus, the duration of hospitalization was not included in logistic regression analysis, although the *P* value of the duration was 0.057. Three risk factors were identified that increased the risk of SPTB <34 weeks: presurgical CL 0 mm (odds ratio (OR): 5.30; 95% confidence interval (CI): 1.58–17.7), a history of SPTB (OR: 4.65; 95% CI: 1.38–15.7), and the presence of sludge (OR: 4.14; 95% CI: 1.20–14.3).

## 4. Discussion

We made a few important observations in patients necessitating emergent cerclage without progesterone administration. SPTB <34 weeks occurred after emergency cerclage in 16% of patients. Three risk factors predicted SPTB <34 weeks: unmeasurable CL (CL 0 mm), a history of SPTB, and the presence of sludge on ultrasound. These results may be similar to those of patients managed with progesterone administration.

A few studies have been reported regarding risk factors for SPTB after cerclage under different pre- or postoperative managements, including no routine tocolysis and progesterone administration. Fuchs et al. [13] reported that four risk factors, a history of PTB, cervical dilatation, membranes bulging into the vagina, and infection (white blood cell ≥13600/mm^3^ or CRP >15 mg/L), are associated with PTB <32 weeks. There are similarities and differences between Fuchs's study and our study. The similarities were that a history of PTB and cervical dilation (“CL = 0 mm”) was associated with PTB. Contrarily, there were some differences in inclusion criteria and risk factors to be examined. He included patients with bulging bag into vagina, whereas we excluded them. Regarding infection, he took WBC and CRP into consideration; however, we evaluated sludge and cervical elastase. Another study concluded that, of patients receiving cerclage during 10–24 weeks, emergent cerclage, a history of conization, and CL <25 mm were risk factors for PTB <32 weeks [14]. Inclusion criteria were also different from our study. The biggest difference was that patients with history-indicated cerclage were included in the previous study [14].

A history of SPTB was a significant risk for SPTB <34 weeks in this study. Numerous studies showed that a history of SPTB is a risk factor for SPTB. Of those, a systematic review and meta-analysis showed that the rate of SPTB (<37 weeks) was high as 30% in 55,197 patients with a history of a preterm singleton livebirth [15]. In addition, two consecutive preterm deliveries increase the risk of subsequent preterm delivery. If a patient has a history of two consecutive very preterm deliveries (SPTB <30 weeks), the risk of SPTB is 57% in the subsequent pregnancy [16]. It is clear that a history of SPTB is one of the most significant risk factors for SPTB based on our results.

An ultrasound-detectable sludge was shown to be a risk for SPTB in this study. A systematic review showed that patients with sludge had higher incidences of SPTB [15, 17]. Recent studies also showed that amniotic fluid sludge is an independent risk factor for PTB [18–20]. Nulliparous patients with CL <30 mm with sludge between 16 and 22 weeks showed an increase in not only PTB <32 weeks (aOR: 2.78 (95% CI: 1.42–5.45)) but also PTB <34 weeks (aOR: 1.85 (95% CI: 1.00–3.44)), revealing that the association between sludge and PTB was greater in earlier gestational weeks [19]. The sludge is considered to reflect a severe intra-amniotic infection-related inflammatory process [21]. The concentration of a proinflammatory cytokine (interleukin-8) in amniotic fluid and presence of histological chorioamnionitis in patients with sludge significantly increased [22]. It is still unknown whether antibiotics decrease PTB in patients with sludge. A recent study showed that antibiotics decreased not only SPTB <34 weeks in patients with shortened CL (<25 mm) but also the incidence of sludge [23]. Inconsistently, antibiotics (azithromycin or moxifloxacin) for patients with sludge did not decrease PTB <37 weeks and <28 weeks compared with those without the antibiotics [24]. Patients with sludge are likely to have significant intrauterine infection, and, thus, it may be difficult for emergent cerclage to show effectiveness.

Unmeasurable CL (CL 0 mm) was also a risk factor for SPTB in this study. Shortened CL is a well-known risk factor for SPTB, and emergent cerclage shows the preventive effect for SPTB in patients with shortened CL. Berghella et al. [25] showed that patients with CL <10 mm, compared with those >10 mm, were less likely to suffer SPTB <35 weeks (30/76 (40%) vs. 29/50 (58%) (RR 0.68 (0.47–0.98)), suggesting that CL <10 mm should be an indication for emergent cerclage in patients even without a history of SPTB. However, emergent cerclage for patients with CL <5 mm did not show the preventive effect significantly (RR 0.79 (0.5–1.23)) [25]. Patients with very short CL such as requiring the replacement of the bag at surgery may have another pathological condition and, thus, emergent cerclage may not show the preventive effect simply. Although it is still unclear how much the cerclage reduces SPTB, unmeasurable CL should be taken into consideration as a high-risk following the emergent cerclage.

Comparing the effectiveness of cerclage or any other treatment with the endpoint being the incidence often causes some difficulty. Our protocol for cerclage may not be completely consistent with the global standard regarding the following two points: (1) Progesterone was not administered, and (2) cerclage was performed for a patient with CL <25 mm even without a history of SPTB. A study involving a large number of showed that cerclage decreased the SPTB incidence for a singleton pregnancy in the presence of a history of SPTB with CL <25 mm, but only in those with a history of SPTB [26]. It remains unknown whether cerclage decreases PTB in patients with CL <25 mm even without a history of SPTB, although several small studies existed. Althuisius et al. reported that emergent cerclage significantly decreased SPTB <34 weeks (1/10 vs. 5/8 patients; *P*=0.04) in a randomized study [27]. On the contrary, a Japanese study also reported that emergent cerclage for patients with ≤CL 25 mm including those without a history of SPTB (*n* = 106) did not decrease SPTB without progesterone administration [28]. A few authors also showed no improvement of perinatal outcomes, including the rate of PTB [25, 29, 30]. Regarding vaginal progesterone administration, a meta-analysis revealed that vaginal progesterone decreased SPTB <33 weeks (14%, RR 0.62, 95% CI, 0.47–0.81) [31]. As described, we did not use progesterone. This may have prompted us to widen the indication of cerclage: we performed cerclage irrespective of the presence/absence of a history of SPTB. In this study, the rate of SPTB <34 weeks after cerclage was 19%. A meta-analysis showed that, of 419 patients with CL < 25 mm without a history of SPTB, the incidence of PTB <34 weeks was 20.1% (45/224) [30]. This rate is comparable to that (16%) in the present study. Considering that the present study included 30 patients with a history of SPTB (considered high-risk), the present results may be satisfactory.

This study had some limitations. First, this was a retrospective analysis, and the study number was relatively small. Second, confounding risk factors could not be completely excluded. For example, intra-amniotic infection can cause SPTB; however, we only checked for the presence or absence of sludge, serum CRP, and cervical elastase. Although interleukin-6 and -8 in amniotic fluid can indicate intra-amniotic infection [32], we did not evaluate them. Third, as described, the management of threatened PTB is not always consistent with the global standard. As stated, we employed long-term tocolysis with ritodrine hydrochloride and/or magnesium sulfate, and systemic antibiotic administration without progesterone. The management background should be taken into account.

## 5. Conclusion

Three risk factors predicted SPTB <34 weeks after emergency cerclage: unmeasurable CL (CL 0 mm), a history of SPTB, and the presence of sludge on ultrasound. SPTB <34 weeks occurred after emergency cerclage in 16% of patients, being comparable with the recent data. This study may have indicated the data of cerclage effectiveness without progesterone, data excluding the effect of progesterone.

## Figures and Tables

**Table 1:** Patients' backgrounds.

Characteristic	*n* = 123
Age (years), median (IQR)	34 (31–36)
<30, *n* (%)	26 (21)
30–34, *n* (%)	46 (37)
35–39, *n* (%)	40 (33)
≥40, *n* (%)	11 (9)
Primiparous, *n* (%)	44 (36)
Pregnancy by ART, *n* (%)	13 (11)
History of CS, *n* (%)	21 (17)
History of myomectomy, *n* (%)	2 (2)
History of cervical cerclage, *n* (%)	7 (6)
History of conization, *n* (%)	2 (2)
History of SPTB	30 (24)
In the 2nd trimester	13 (11)
In the 3rd trimester	17 (14)
Slight bleeding at admission, *n* (%)	7 (6)
Presurgical CL (mm), median (IQR)	16 (8–21)
Presurgical CL <10 mm, *n* (%)	35 (28)
Presurgical CL 0 mm, *n* (%)	19 (15)
Presence of sludge in ultrasound, *n* (%)	24 (16)
Cervical elastase positive, *n* (%)^*∗*^	20 (23)
Presence of *Lactobacillus*, *n* (%)^∗∗^	89 (72)
Presence of ureaplasma, *n* (%)^∗∗∗^	42 (34)
Presurgical C-reactive protein (mg/dL), median (IQR)	0.21 (0.10–0.37)

Procedure of cerclage	
McDonald	123 (100)
Replacement of prolapsed membranes at surgery, *n* (%)	22 (18)

ART, assisted reproductive technology; CL, cervical length; IQR, interquartile range; SPTB, spontaneous preterm birth. ^*∗*^Not examined in 37 patients, ^∗∗^not examined in 2 patients,^∗∗∗^ not examined in 28 patients.

**Table 2:** Perinatal outcome of patients with cervical cerclage.

	*n* = 123
GA at delivery, *n* (%)	
33 + 6 or less weeks	20 (16)
34+0–36 + 6 weeks	24 (20)
≥37 weeks	79 (64)
GA at delivery with 33 + 6 or less weeks, *n* (%)	
<22 weeks	4 (3)
22+0201325 + 6 weeks	6 (5)
26+0–29 + 6 weeks	3 (2)
30+0–33 + 6 weeks	7 (6)
Duration between operation and discharge (days), median (IQR)^*∗*^	48 (14–96)
Birth weight (grams), median (IQR)	2706 (2340–3030)
Apgar score 1 mins, mean ± SD	7.44 ± 1.74
Apgar score 5 mins, mean ± SD	8.64 ± 1.01
NICU admission, *n* (%)^∗∗^	51 (41)

GA, gestational age; IQR, interquartile range; NICU, neonatal intensive care unit; SD, standard deviation, ^*∗*^duration between operation and delivery, if delivery occurred during hospitalization. ^∗∗^Four abortive fetuses were excluded.

**Table 3:** Univariate analysis of risk factors among preterm deliveries less than 34 weeks.

	<34 weeks (*n* = 20)	≥34 weeks (*n* = 103)	*P*-value
Age, median (IQR)	34 (32–36)	33 (30–37)	0.488
Primiparous, *n* (%)	9 (45)	35 (34)	0.445
Pregnancy by ART, *n* (%)	1 (5)	13 (13)	0.691
Presence of cervical polyp, *n* (%)	1 (5)	7 (7)	1.000
History of cerclage, *n* (%)	0 (0)	7 (7)	0.597
History of CS, *n* (%)	1 (5)	20 (19)	0.192
History of myomectomy, *n* (%)	1 (5)	1 (1)	0.300
Slight bleeding at admission, *n* (%)	2 (10)	5 (5)	0.318
Presurgical CL, mm (IQR)	8 (0–21)	16 (10–21)	0.082
Presurgical CL 0 mm, *n* (%)	8 (40)	12 (12)	0.003
Presence of sludge in ultrasound, *n* (%)	8 (40)	16 (16)	0.029
Presence of *Lactobacillus*	14 (70)	75 (73)	0.782
Presence of ureaplasma	7 (35)	35 (34)	1.000
Cervical elastase positive, *n* (%)	4 (20)	16 (16)	0.272
GA at emergency cerclage, week (IQR)	21 (18–24)	22 (20–23)	0.377
History of SPTB, *n* (%)	8 (40)	22 (21)	0.091
History of cerclage, *n* (%)	1 (5)	9 (9)	0.687
Duration between cerclage and discharge (days), median (IQR)^*∗*^	35 (21–84)	76 (13–98)	0.057

ART, assisted reproductive technology; CL, cervical length; CS, cesarean section; GA, gestational age; IQR, interquartile range; SPTB, spontaneous preterm birth. ^*∗*^Long-term tocolysis and hospitalization management was employed for the majority of the patients. Thus, the duration of hospitalization was not included in logistic regression analysis.

**Table 4:** Multivariate analysis of risk factors among SPTBs less than 34 weeks.

	Odds ratio (95% CI)	*P* value
Presurgical CL 0 mm	5.30 (1.58–17.7)	0.007
History of SPTB	4.65 (1.38–15.7)	0.013
Presence of sludge in ultrasound	4.14 (1.20–14.3)	0.025

CL; cervical length, SPTB, spontaneous preterm birth.

## Data Availability

The data used to support the findings of this study are available from the corresponding author upon request.
